# Age related non-type 2 inflammation and its association with treatment outcome in patients with chronic rhinosinusitis with nasal polyp in Korea

**DOI:** 10.1038/s41598-022-05614-z

**Published:** 2022-01-31

**Authors:** Jeong-Whun Kim, Tae-Bin Won, Hyunjun Woo, Seung Koo Yang, Chayakoen Phannikul, Seung Ah Ko, Hyojin Kim, Chae-Seo Rhee, Sung-Woo Cho

**Affiliations:** 1 Department of Otorhinolaryngology-Head and Neck Surgery, Seoul National University Bundang Hospital, Seoul National University College of Medicine, 82 Gumi-ro 173beon-gil, Bundang-gu, Seongnam, 13620 Korea; 2 Department of Otorhinolaryngology-Head and Neck Surgery, Seoul National University Hospital, Seoul National University College of Medicine, Seoul, Korea; 3 Department of Pathology, Seoul National University Bundang Hospital, Seoul National University College of Medicine, Seongnam, South Korea

**Keywords:** Diseases, Medical research

## Abstract

This study aimed to investigate the effect of age in patients with chronic rhinosinusitis with nasal polyp (CRSwNP). 269 patients were divided into eosinophilic and non-eosinophilic groups based on tissue eosinophilia, defined by eosinophils accounting for more than 20% of the total inflammatory cells. Patients were then further divided into younger and older groups based on the age of 35 years. Clinical characteristics including blood eosinophil, Lund Mackay score, and modified Lund-Kennedy (mLK) scores were compared. Levels of 14 cytokines from nasal tissues of an additional 78 patients were analyzed. Tissue eosinophilia was significantly associated with age and the proportion of non-eosinophilic CRSwNP was significantly higher in younger patients as compared to older patients (79.2% vs 56.6%). There was no difference in clinical characteristics and cytokine levels between the younger and older patients with eosinophilic CRSwNP. In contrast, in patients with non-eosinophilic CRSwNP, younger patients had significantly lower preoperative blood eosinophils and higher mLK scores at three and six months, postoperatively, compared to older patients. Alpha-1 antitrypsin and IL-5 levels were significantly lower in younger patients than in older patients in non-eosinophilic CRSwNP. This study suggests a potential association between age, non-type 2 inflammation and treatment outcome in CRSwNP.

## Introduction

Chronic rhinosinusitis with nasal polyps (CRSwNP) is a common inflammatory upper airway disorder that affects approximately 1–4% of the population worldwide^1,2^. A significant proportion of patients require surgical treatment, such as functional endoscopic sinus surgery, which is the current standard. However, CRSwNP is a heterogeneous disorder that is composed of many disease subtypes. Patients with tissue eosinophilia and high IL-5 levels have a higher prevalence of comorbid asthma and difficult-to-treat cases^1,3–5^. For difficult-to-treat cases, additional surgical procedures, or medical treatment, such as biologics, may be required^6–8^. Therefore, it is important to identify patients who may require additional therapy in conjunction with standard care. The Japanese Epidemiology Survey of Refractory Eosinophilic Chronic Rhinosinusitis Study (JESREC) demonstrated that tissue eosinophilia is an important marker for polyp recurrence^9^. This study proposes a novel scoring system using routinely available clinical markers. A high JESREC score indicates the presence of eosinophilic CRS, and patients are expected to have higher recurrence rates after standard surgical treatment.

Non-eosinophilic polyps are diagnosed in a significant proportion of Asian populations. Studies have demonstrated refractory cases among patients with non-eosinophilic polyps that may not be reliably predicted by the JESREC scoring system^4,10^. Very little is known about the risk factors associated with disease refractoriness in non-eosinophilic polyps.

We previously employed unsupervised cluster analysis to categorize heterogeneous CRSwNP subtypes using routinely available clinical markers. Our previous studies have shown that treatment outcomes can vary with the age of patients with non-eosinophilic polyps^11^. Patients aged less than 35 years tended to have worse outcomes, as manifested by higher revision surgery rate. Therefore, age may be an important clinical factor associated with treatment outcomes in CRSwNP. The goal of this study was to evaluate the association between age and the clinical and immunological parameters of CRSwNP.

## Materials and methods

### Study population

This study retrospectively analyzed the medical records of patients with CRSwNP who underwent bilateral functional endoscopic sinus surgery (FESS) at Seoul National University Bundang Hospital (Bundang, South Korea) between June 2017 and January 2020; a period that had not been previously analyzed. Patients of all ages with refractory primary bilateral CRSwNP were analyzed. Diagnosis of CRSwNP was made based on the criteria described by the European position paper on rhinosinusitis with nasal polyps^12^. Patients who had undergone FESS before June 2017 or who had been treated with preoperative systemic corticosteroids within the four‐week surgery period were excluded. Patients with unilateral disease, fungal disease, antrochoanal polyps, cystic fibrosis, primary ciliary dyskinesia, or other tumorous conditions, such as sinonasal inverted papilloma, were excluded. Only patients of Asian ethnicity were included in the study.

Two independent cohorts of patients were analyzed. The first cohort (cohort 1) comprised 269 patients with primary bilateral CRSwNP who underwent FESS during the study period and had been followed up for at least three months after the surgery. The second cohort (cohort 2) comprised 65 primary bilateral CRSwNP patients and 13 controls, both of which underwent immunological analysis for tissue cytokine levels and evaluation of clinical characteristics. The controls were patients who underwent endoscopic skull base surgery without any evidence of sinonasal disease. All subjects who underwent tissue analysis provided written informed consent for participation in the study. This study was approved by the Institutional Review Board of the Seoul National University Bundang Hospital (B-2009–636-302). All methods were performed in accordance with the relevant guidelines and regulations.

### Analysis of the perioperative clinical parameters

For all patients, Lund-Mackay (LM) scoring was performed using a CT scan^13^. This grades each sinus separately on the 0 to 2 scale depending on whether the sinuses are clear, partially, or completely opacified. The sum of the LM scores for the right and left nasal cavities were then, calculated. The presence of asthma was assessed by the institution's allergist after thorough review of previous medical history and lung function tests: spirometry, methacholine challenge test, or bronchodilator response test. A complete blood cell count with differential was performed within four weeks prior to surgery to check the peripheral blood eosinophil count. Nasal polyp tissues were obtained during FESS for histopathological analysis. Postoperative specimens were reviewed by pathologists at our institution. Pathological findings were reported as “eosinophilic” or “non‐eosinophilic.” Tissue eosinophilia was considered when eosinophils accounted for more than 20% of the total inflammatory cells at 400X magnification^14^. Nasal polyps were defined as eosinophilic or non-eosinophilic according to tissue eosinophilia status. Smoking status was also collected during the study period and was reported as never smoker or current/ex-smoker.

### Patient classification

Patients were classified according to their tissue eosinophilia status. They were further divided as younger or older by the age above or below 35 years; NE-Y (non-eosinophilic CRSwNP with younger age), NE-O (non-eosinophilic CRSwNP with older age), E-Y (eosinophilic CRSwNP with younger age) and E-O (eosinophilic CRSwNP with older age). The cutoff value of 35 years was based on a previously described decision tree, which was constructed after cluster analysis using a patient's clinical features, including age, peripheral blood eosinophil, tissue eosinophilia, Lund‐Mackay computed tomography (CT) scores, ratio of the CT scores for the ethmoid sinus and maxillary sinus, and comorbid asthma^11^.

### Cytokine measurement from tissue homogenates

Tissues were harvested from nasal polyps in CRSwNP patients during FESS and the ethmoid sinus mucosa in the controls without any evidence of sinonasal disease during the endoscopic endonasal approach for pituitary adenoma or skull base tumors. Tissue samples were snap-frozen in liquid nitrogen and stored at − 80 °C, thawed at room temperature, and homogenized with a mechanical homogenizer. After homogenization, the suspensions were centrifuged at 4000 rpm for 20 min at 4 °C, and the supernatants were separated and stored at 80° C for further analysis. The samples were thawed and vortexed at room temperature to ensure adequate mixing. Protein concentrations of tissue extracts were determined using Pierce 660 nm Protein Assay Kits (Thermo Fisher Scientific Inc., Rochester, NY, USA). Tissue homogenates were then assayed using the multiple cytokine analysis kits: macrophage inflammatory protein (MIP)-1β/CCL4, interferon (IFN)-γ, interleukin (IL)-1β/IL-1F2, IL-5, IL-6, IL-8/CXCL8, IL-12 (p70), IL-33, tumor necrosis factor (TNF)-α (Luminex Human Magnetic Assay (10-Plex) (LXSAHM-10; R&D Systems, Minneapolis, MN, USA), myeloperoxidase (MPO) (Luminex Human Magnetic Assay (LXSAHM-01); R&D Systems), TGFβ1 (TGFBMAG-64K-01; Millipore, Billerica, MA, USA), α1 antitrypsin (A1AT) (HNDG2MAG-36K-01; Millipore), human neutrophil elastase (HNE) (HSP3MAG-63K-01; Millipore), and IL-22 (HTH17MAG-14K-01; Millipore). Data were collected using a Luminex 100 reader (Luminex, Austin, TX, USA). Data analysis was performed using MasterPlex QT version 2.0 software (MiraiBio, Alameda, CA, USA). All assays were performed in duplicate, according to the manufacturer’s protocol. All protein levels in the tissue homogenates were normalized to total protein concentration. The sensitivity for each cytokine were as follows :macrophage : MIP-1β/CCL4 (5.8 pg/ml), IFN-γ (0.4 pg/ml), IL-1β/IL-1F2 (0.8 pg/ml), IL-5 (0.5 pg/ml), IL-6 (1.7 pg/ml), IL-8/CXCL8 (1.8 pg/ml), IL-12 (p70) (20.2 pg/ml), IL-33 (1.8 pg/ml), TNF-α (1.2 pg/ml), MPO (26.2 pg/ml), TGFβ1 (11.4 pg/ml), α1 anti-trypsin (0.085 ng/ml), human neutrophil elastase (HNE) (6.2 pg/ml) and IL-22 (0.032 pg/ml). Values less than the detection limit were considered as half of the lower limit^5^.

### Evaluation of treatment outcomes

After standard FESS, oral antibiotics were routinely prescribed for five-seven days, and intranasal corticosteroid spray was used afterward. Patients were encouraged to perform saline nasal irrigation for three months. Endoscopic findings were evaluated three months, six months, and one year postoperatively using a modified Lund-Kenney (mLK) score^15^ from the physician’s report. The mLK scores assesses edema, discharge on the 0 to 2 scale for each parameter, for a total possible score of 6. The scores on both sides were then summed. Short courses of antibiotics or systemic corticosteroids were prescribed when necessary. In such cases, endoscopic examinations were re-evaluated after two weeks and were used for analysis.

### Statistical analysis

Differences between groups were analyzed using the Kruskal–Wallis tests with Dunn multiple comparisons tests or Mann–Whitney *U*-test, after confirmation of a non-parametric distribution, by the Shapiro tests for continuous variables. To reduce the false discovery rate, the method of Benjamini and Yekutieli was used^16^ Chi-square or Fisher’s exact test tests were performed for categorical variables. Results are presented as mean ± standard deviation (SD) or median with interquartile range (IQR). Spearman correlation coefficients were determined to assess the relationship between cytokine levels and age. Cytokine data that significantly correlated with age were displayed using locally weighted scatterplot smoothing regression curve by R 3.4.2 software (R Foundation for Statistical Computing; http://www.r-project.com). Statistical analyses were performed using IBM SPSS Statistics version 22.0 software (; IBM Corp., Armonk, NY, USA) and R 3.4.2 software. Statistical significance was set at *p* < 0.05.

## Results

### General characteristics

**Figure 1:**
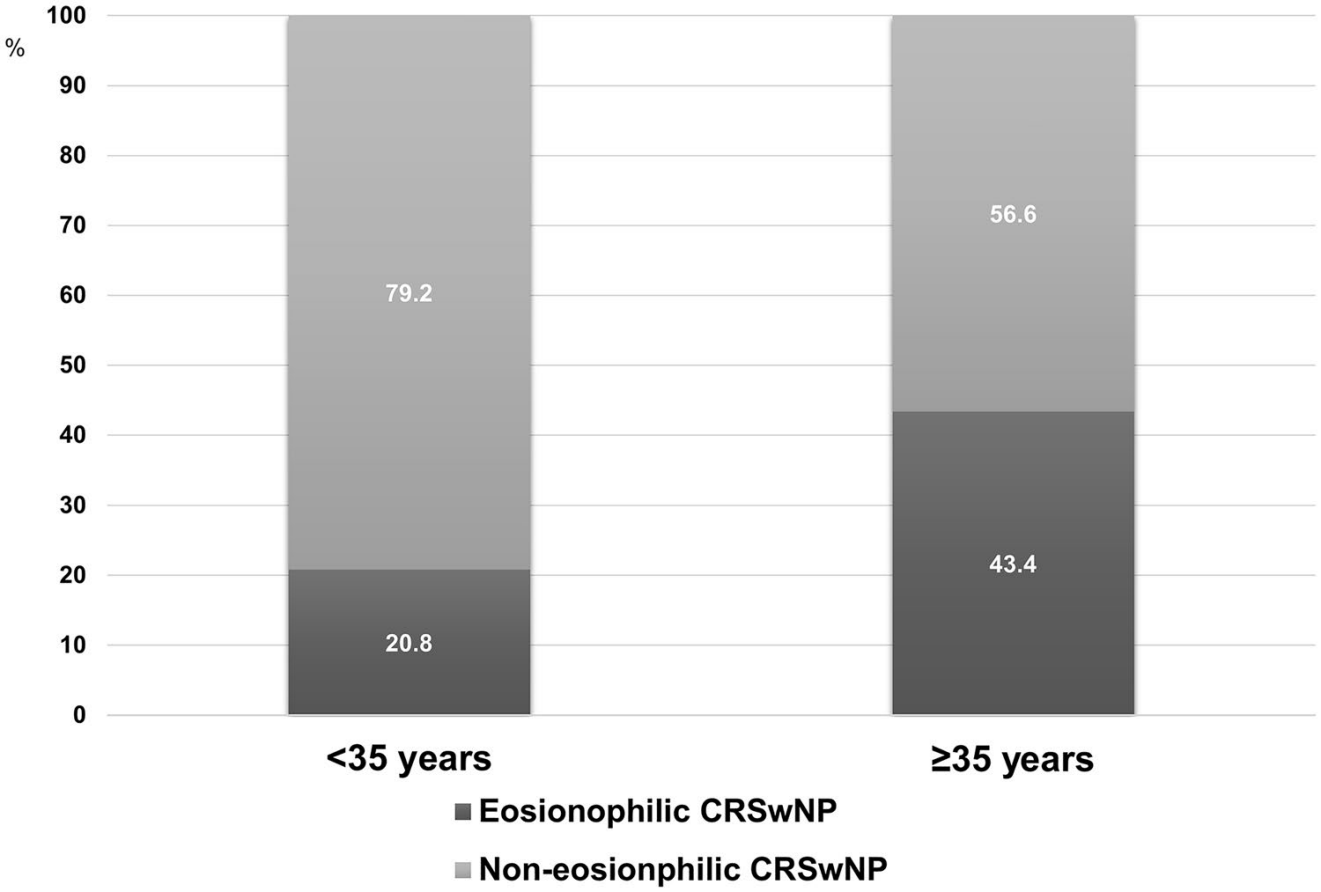
Distribution of eosinophilic and non-eosinophilic chronic rhinosinusitis with nasal polyp (CRSwNP) among younger (< 35 years) and older age groups (≥ 35 years).

Cohort 1 included 106 patients with eosinophilic CRSwNP and 163 patients with non-eosinophilic CRSwNP. Among patients with eosinophilic CRSwNP, 10 (9.4%) were younger (E-Y) and 96 (90.6%) were older (E-O). Of the non-eosinophilic CRSwNP patients, 38 (23.3%) were of younger age (NE-Y) and 125 (76.7%) were of older age (NE-O). There was a strong association between age group and tissue eosinophilia status (p = 0.004). The prevalence of non-eosinophilic CRSwNP was significantly higher in younger patients than in older patients (79.2% vs. 56.6%) (Fig. 1).

The clinical characteristics of cohort 1 are summarized in Table 1. Comorbid asthma (20.8% vs 11.7%, p = 0.042) and blood eosinophils (8.0 ± 4.1% vs 4.9 ± 4.7%, p < 0.001) were significantly higher in patients with eosinophilic CRSwNP compared to that of non-eosinophilic CRSwNP. There was no significant difference in clinical characteristics between E-Y and E-O. However, patients with NE-O had a significantly higher level of blood eosinophils compared to that with NE-Y (5.3 ± 5.0% vs 3.5 ± 3.2%, p = 0.016). Other clinical characteristics were not significantly different between NE-Y and NE-O.

**Table 1:** Characteristics and treatment outcome.

Clinical characteristics	Overall			Eosinophilic CRSwNP			Non-eosinophilic CRSwNP		
	Eosinophilic CRSwNP (N = 106)	Non-eosinophilic CRSwNP (N = 163)	P-value	Younger age (N = 10)	Older age (N = 96)	*P*-value	Younger age (N = 38)	Older age (N = 125)	P-value
Age (years)	49.6 ± 12.0	47.3 ± 17.9	0.835	27.1 ± 7.6	60.0 ± 9.8	< 0.001	20.5 ± 7.2	55.5 ± 10.6	< 0.001
LM score	16.9 ± 3.4	17.5 ± 4.6	0.54	16.5 ± 3.3	16.9 ± 3.5	0.724	18.5 ± 5.5	17.2 ± 4.2	0.197
Blood eosinophil (%)	**8.0 ± 4.1**	**4.9 ± 4.7**	** < 0.001**	8.9 ± 3.7	7.9 ± 4.1	0.442	**3.5 ± 3.2**	**5.3 ± 5.0**	**0.016**
Asthma (%)	**20.8**	**11.7**	**0.042**	0	22.9	0.117	7.9 (3/38)	12.0 (15/125)	0.480
Smoking (%)	56.5	45.9	0.110	59.1	55.7	0.780	47.2	45.5	0.853
LK 3	**1.1 ± 1.2** **(103/106)**	**1.9 ± 2.0** **(155/163)**	**0.008**	1.7 ± 1.5	1.1 ± 1.2	0.118	**2.9 ± 2.5** **(35/38)**	**1.5 ± 1.7** **(120/125)**	** < 0.001**
LK 6	1.4 ± 1.2 (25/106)	1.8 ± 1.9 (53/163)	0.629	2.0 ± 2.0	1.3 ± 1.1	0.509	**2.5 ± 1.9** **(17/28)**	**1.5 ± 1.8** **(36/125)**	**0.045**
LK 12	1.0 ± 1.4 (23/106)	2.0 ± 2.4 (42/163)	0.088	0.5 ± 0.7	1.0 ± 1.4	0.901	2.0 ± 2.7 (12/38)	2.0 ± 2.3 (30/125)	0.528

CRSwNP: chronic rhinosinusitis with nasal polyps, LM: Lund Mackay, LK: Lund Kennedy. The results for continuous variables are presented as means with standard deviations. The age cut-off was 35 years. Values in brackets indicate the proportion of patients who were checked for postoperative outcomes. Significance values are in bold.

### Treatment outcomes

When it comes to treatment outcome, post-operative mLK scores three months were significantly lower in patients with eosinophilic CRSwNP compared to those with non-eosinophilic CRSwNP (1.1 ± 1.2 vs 1.9 ± 2.0, p = 0.008). However, six months and twelve months post-operative mLK scores were not significantly different.

In eosinophilic CRSwNP, there was no significant difference in outcomes between E-Y and E-O. However, in non-eosinophilic CRSwNP, compared to NE-O, NE-Y had higher mLK scores measured at three months (1.5 ± 1.7 vs. 2.9 ± 2.5, respectively, p < 0.001) and six months (1.5 ± 1.8 vs. 2.5 ± 1.9, respectively, p = 0.045) postoperatively. NE-Y had a significantly higher mLK score 3 months postoperatively compared to E-O (p < 0.001). (Table 1).

### Immunological characteristics

Cohort 2 comprised 39 patients with eosinophilic CRSwNP, 26 patients with non-eosinophilic CRSwNP and 13 controls. Clinical characteristics of cohort 2 are described in S1 Table. Age, male to female ratio, and LM scores were not significantly different between patients with eosinophilic and non-eosinophilic CRSwNP. Comorbid asthma (p = 0.042) and blood eosinophils (p < 0.001) were significantly higher in patients with eosinophilic CRSwNP compared to those with non-eosinophilic CRSwNP or controls.

**Figure 2:**
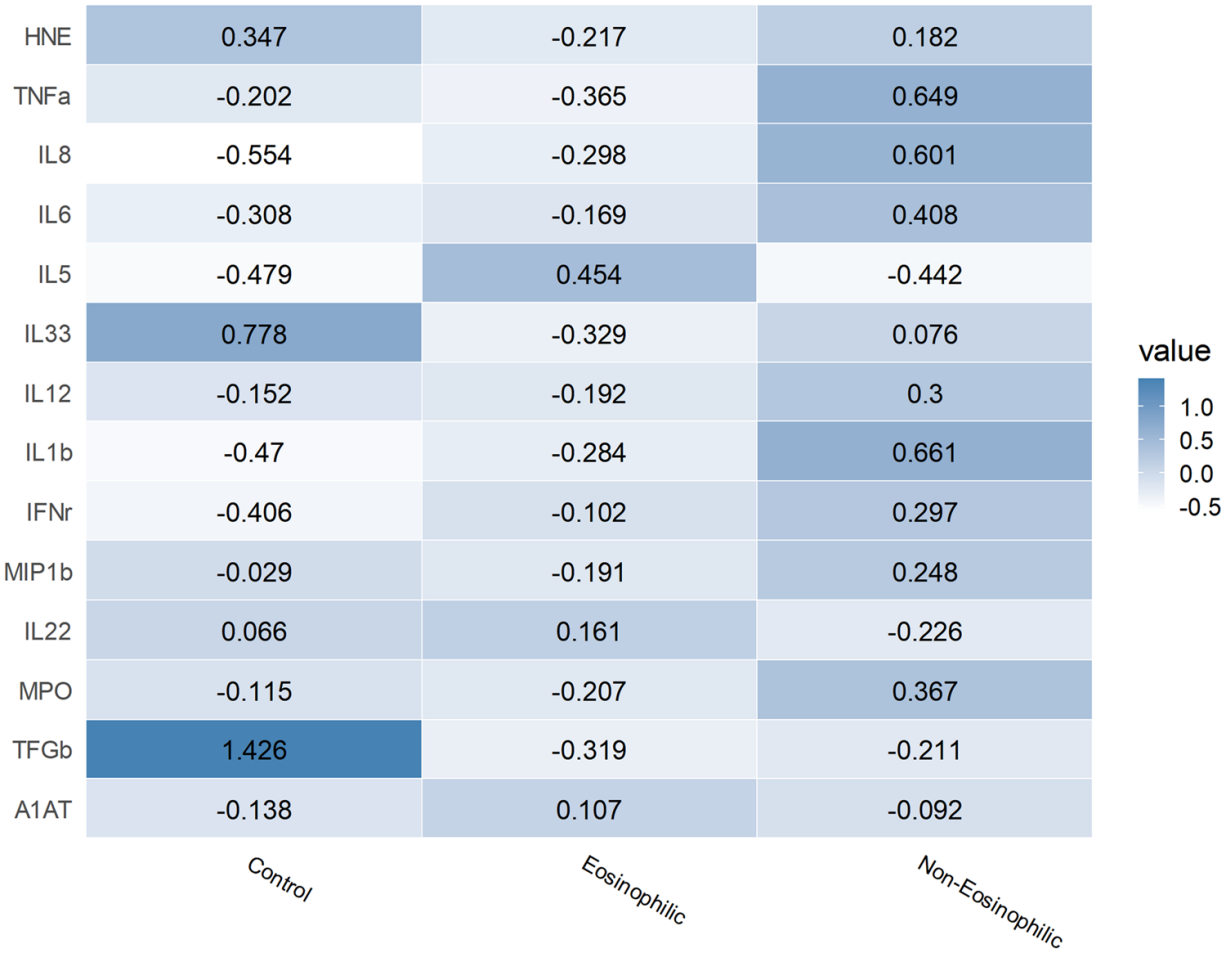
Heatmap of protein concentrations among patient classes. The color bar presents Z-scores of tissue concentration for each measured cytokine.

A heatmap of protein concentrations among patient groups is shown in Fig. 2. Grossly looking, control tissue, eosinophilic polyp, and non-eosinophilic polyp are clearly separated. Cytokine levels of TGF-ß, IL-1ß, IL-33, IL-5, IL-6, IL-8, and TNF-α were significantly different among groups. Post hoc comparison revealed a significant difference in the levels of IL-1ß and IL-5, IL-6, IL-8, and TNF-α between eosinophilic and non-eosinophilic polyp (S2 Table).

**Figure 3:**
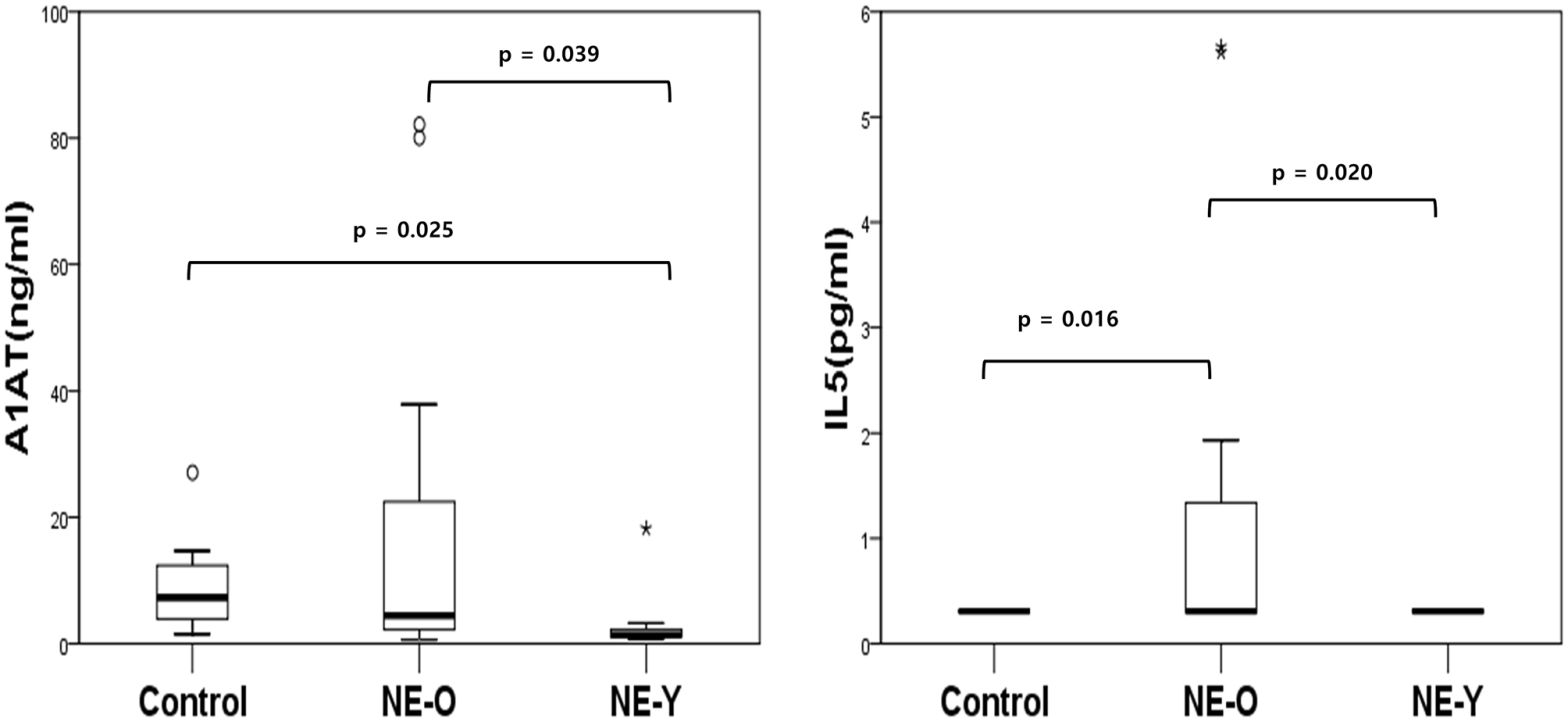
Inflammatory mediators in NE-O and NE-Y. Data were evaluated by Kruskal–Wallis test and Dunn multiple comparison test. Bars indicate p < 0.05 after post hoc Dunn’s test. NE-Y: Non-eosinophilic polyp with age under 35 years, NE-O: Non-eosinophilic polyp with age over 35 years, A1AT: Alpha 1 antitrypsin.

**Figure 4:**
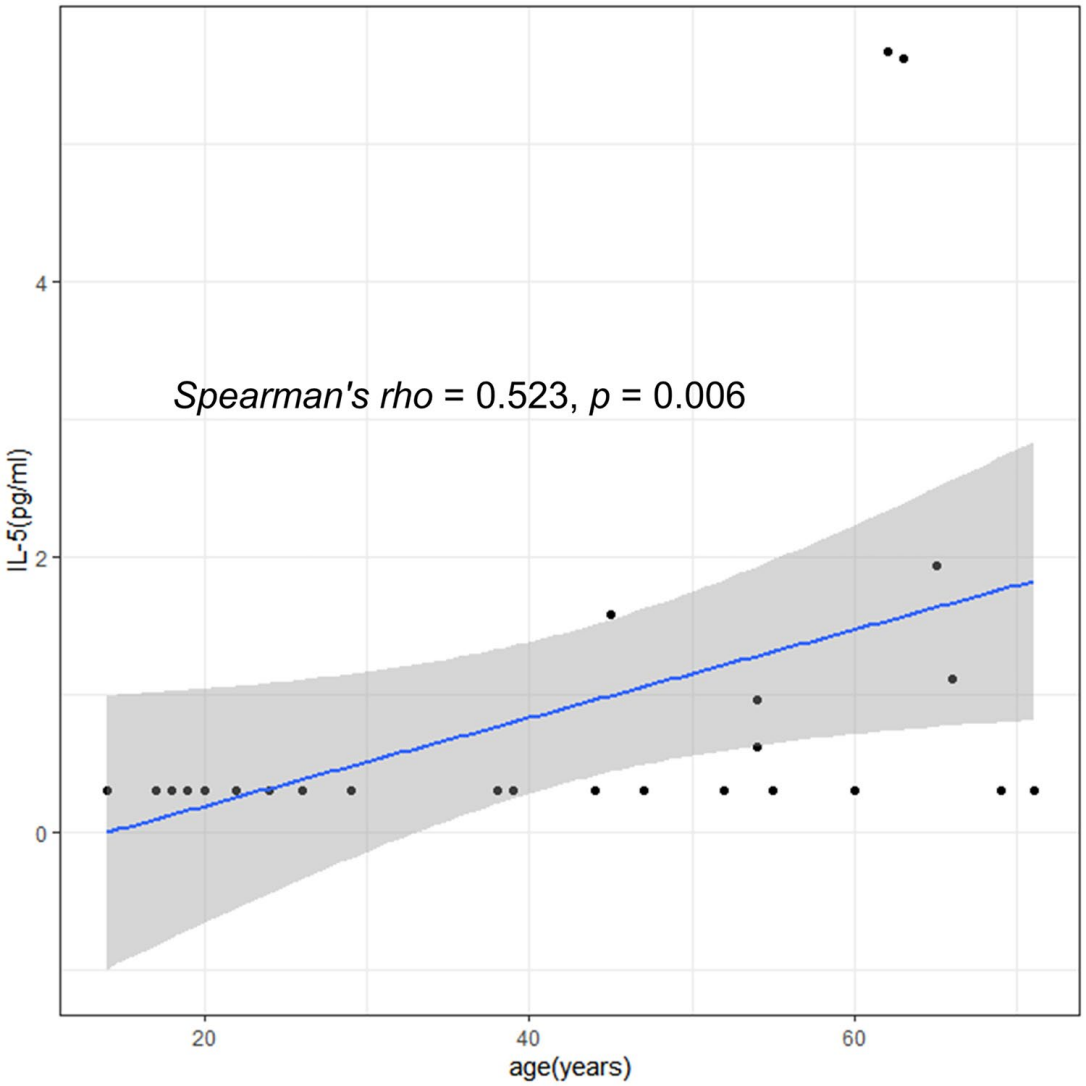
Correlation bewteen tissue IL-5 level and age in patients with non-eosinophilic chronic rhinosinusitis with nasal polyp.

Among patients with eosinophilic CRSwNP,6 patients (15.4%) were younger (E-Y) and 33 patients (84.6%) were older (E-O), while in patients with non-eosinophilic CRSwNP, 10 (38.5%) were younger (NE-Y) and 16 (61.5%) were older (NE-O). Cytokine measurements were not statistically different between E-Y and E-O (S3 Table). In cases of non-eosinophilic polyps, A1AT and IL-5 levels were significantly lower in NE-Y than in NE-O (Fig. 3). The cytokine measurements of non-eosinophilic polyps are summarized in Table 2. Of the 14 cytokines, IL-5 showed a positive correlation with age (Spearman's rho = 0.523, *p* = 0.006, Fig. 4) in non-eosinophilic polyps. However, there was no significant relationship between cytokine levels and age in the eosinophilic polyp group (S4 Table).

**Table 2:** Comparison of immunological characteristics of non-eosinophilic polyps with age under 35 years and over 35 years in cohort 2.

Cytokine	Control (N = 11)	Non-eosinophilic polyp , younger age (N = 10)	Non-eosinophilic polyp, older age (N = 16)	*P*-value
A1AT* (ng/ml)	7.292 (10.890)	1.344 (1.419)	4.412 (25.458)	**0.042**
TGF-ß (pg/ml)	180.712 (185.965)	55.043 (27.409)	56.333 (47.215)	**0.035**
MPO (pg/ml)	7550.833 (38,901.892)	2090.909 (35,671.620)	3878.053 (90,536.668)	0.705
IL-22 (pg/ml)	1.995 (7.038)	0.567 (3.163)	2.208 (4.080)	0.466
MIP1ß (pg/ml)	91.565 (68.393)	94.479 (47.494)	125.557 (66.891)	0.242
IFN-γ (pg/ml)	0.314 (3.059)	1.029 (4.512)	4.798 (8.230)	0.092
IL-1ß (pg/ml)	0.001 (0.459)	3.3216 (5.522)	2.269 (6.350)	0.047
IL-12 (pg/ml)	0.118 (0.000)	0.118 (3.309)	0.118 (0.089)	0.175
IL-33 (pg/ml)	1260.871 (768.614)	823.154 (925.408)	1171.371 (784.733)	0.358
IL-5* (pg/ml)	0.306 (0.000)	0.306 (0.000)	0.306 (1.151)	**0.019**
IL-6 (pg/ml)	1.624 (2.012)	16.949 (25.258)	8.627 (26.802)	**0.019**
IL-8 (pg/ml)	34.912 (18.872)	173.579 (676.759)	206.911 (318.642)	**0.014**
TNF-α (pg/ml)	0.964 (0.719)	1.245 (1.234)	1.173 (1.084)	0.242
HNE (ng/ml)	8762.298 (15,155.938)	5363.075 (2722.129)	5431.329 (3171.724)	0.705

Values are presented as medians with interquartile ranges (IQR). A1AT, alpha 1 antitrypsin; TGF, *transforming growth factor;* MPO, myeloperoxidase; MIP, macrophage inflammatory protein; IFN, interferon; IL, interleukin; TNF, tumor necrosis factor; HNE, human neutrophil elastase. * *P*-value < 0.05, for non-eosinophilic polyp < 35 years vs. non-eosinophilic polyp ≥ 35 years by Kruskal–Wallis test with Dunn multiple comparison. Significance values are in bold.

There were no significant differences in clinical characteristics and treatment outcomes between NE-Y and NE-O nor E-Y and E-O in cohort 2 (data not shown).

## Discussion

In current study, patients were first categorized according to tissue eosinophilia, defined as eosinophils accounting for more than 20% of total inflammatory cells when viewed at high magnification. Although this method is only semiquantitative and may depend on the pathologist’s subjective judgment, it is intuitive and swift. In this study, we analyzed our results from an immunological perspective with a panel of multiple cytokines. Eosinophilic polyps had elevated levels of IL-5 (type 2 inflammation), whereas non-eosinophilic polyps had elevated levels of IL-1b, IL-6, IL-8, and TNF-α (non-type 2 inflammation). Therefore, defining tissue eosinophilia based on histological analysis seems to incorporate immunological characteristics as well.

Although immunologically distinct, tissue eosinophilia status alone does not seem to incorporate prognosis, since treatment outcomes of patients with eosinophilic CRSwNP were similar to those with non-eosinophilic CRSwNP^14^. Therefore, for Koreans population, the dichotomous classification into eosinophilic and non-eosinophilic subtypes is not enough and subclassification beyond tissue eosinophilia is necessary. Our previous study demonstrated that several phenotypes of CRSwNP can be classified using routinely available clinical markers. According to our previous study, among patients with non-eosinophilic polyps, patients less than 35 years of age had worse outcomes compared to patients older than 35 years of age. Therefore, the goal of this study was to validate whether age can be used as a factor that could clinically and immunologically differentiate patients with CRSwNP.

First, age seems to be associated with the tissue eosinophilia status. In patients less than 35 years of age, almost 80% were non-eosinophilic CRSwNP suggesting that CRSwNP in younger age is more associated with non-type 2 inflammation. In addition, within patients with non-eosinophilic CRSwNP, younger patient (NE-Y) seems to be clinically and immunological distinct compared to older patients (NE-O).

Compared to NE-O, NE-Y was characterized by lower levels of IL-5 and A1AT. This suggests an age-dependent association for IL-5 and A1AT levels. Although measured from healthy adults, serum concentrations of A1AT is known to increase with age^17^. Type 2 mediators, including IL-5 and chemokine (CC motif) ligand 24 (CCL-24), have also been shown to increase with age in patients with non-eosinophilic CRSwNP^18^. An unpublished study from Korea demonstrated the duration of the disease in non-eosinophilic CRSwNP is correlated with the tissue level of IL-5^19^ suggesting that prolonged disease duration in NE-O may explain higher level of IL-5. However, this must be further validated as we have not measured disease duration in the current study.

From clinical perspective, blood eosinophils were significantly lower in NE-Y than in NE-O which can be partially explained by the higher level of IL-5 in NE-O patients. In addition, mLK scores measured at three months and six months postoperatively were significantly higher in NE-Y patients than in NE-O patients. As mLK score reflects disease outcome after the surgery^15^, NE-Y patients had worse postoperative short-term outcomes compared to NE-O patients, which is in accordance with the results of our previous study^11^. Therefore, decreased levels of IL-5 and A1AT in the tissue samples and worsening short-term outcomes result in NE-Y being a distinguishing phenotype in CRSwNP.

In the current study, patients were treated with short courses of systemic corticosteroid therapy when necessary. As blood or tissue eosinophil is known to be associated with corticosteroid response^20–22^, better endoscopic scores in NE-O patients than in NE-Y patients may have been derived from higher blood eosinophil in NE-O patients. Similarly, this may also explain a significantly lower endoscopic scores in patients with eosinophilic CRSwNP than in patients with non-eosinophilic CRSwNP at post operative 3 months. Poor treatment outcomes may also be explained by the low levels of IL-5 and A1AT in NE-Y. In non-eosinophilic CRSwNP, one of the possible factors responsible for disease refractoriness is tissue neutrophilia^4^. IL-5, which is a typical type 2 inflammatory cytokine, is known to suppress neutrophil activity^23^. Α1ΑΤ exerts its anti-inflammatory action by inhibiting inflammatory HNE, a serine protease stored in the azurophilic granules of neutrophils and released during neutrophil degranulation^24^. Low Α1ΑΤ is known to be associated with disease refractoriness and tissue neutrophilia in nasal polyps^10^. Therefore, characteristics of NE-Y may be associated with increased neutrophilic inflammation. However, this needs to be further evaluated since there was no significant difference in expression level neutrophilic markers, such as MPO, HNE, and IL-8 between NE-Y and NE-O.

There was a significant increase in proportion of eosinophilic CRSwNP in older patients compared to younger patients (20.8% to 43.4%). This may also mean that type 2 inflammation is more associated with older age that there is a higher proportion of eosinophilic CRSwNP compared to younger patients. Age association to eosinophilic CRSwNP can be partially explained by physiological and functional changes in the nasal mucosa, epithelial barrier dysfunction, or interaction with microbiome manifested as IgE responses to Staphylococcal enterotoxin B which is also known to be associated with adult onset asthma^25–27^.

However, there were no significant clinical or immunological differences between young and old-aged eosinophilic polyps. Our study showed somewhat contradictory results from other studies. Other studies had shown that elderly patients with CRS showed significantly higher CT scores, which were associated with nasal polyps^28,29^. From immunological perspective, age affected tissue cytokine profile differently in eosinophilic polyps. Among eosinophilic polyps, levels of IL-17 and chemokine C-X-C motif ligand (CXCL1), both of which are type 3 cytokines related to neutrophilic inflammation, positively correlated with age^30^. The discrepancy between current study and others may be due to arbitrarily determined age cutoff values among patients with eosinophilic polyps and the limited number of patients, especially those with younger age.

Our study has several limitations. This is not a longitudinal study and demonstrates only a cross-sectional association of age and disease endotype. Therefore, our study does not suggest that disease endotype may change over time as demonstrated in other study^31^. Postoperative endoscopy scores were the only outcome measured in our study. Since the endoscopy scores tend to reach a nadir around three to six months postoperatively and rise afterward^32^, a longer follow-up duration is necessary to observe differences between the patient groups. Also, as this is a single center study, there may be a risk of selection bias. Therefore, a larger cohort, perhaps a multicenter study, with long-term follow-up is warranted.

In summary, age is associated the tissue eosinophilia status in CRSwNP. Especially, for younger patients, there was a significantly higher proportion of non-eosniphilic CRSwNP. Age appears to have a significant effect in patients with non-eosinophilic CRSwNP. Younger patients had worse treatment outcomes and were characterized by low IL-5 and A1AT compared to older patients with non-eosinophilic CRSwNP. Therefore, age may be an important parameter for phenotyping CRSwNP.

## Supplementary Information

Supplementary Information 1.

Supplementary Information 2.

## Data Availability

The data cannot be publicized for legal reasons in Korea.
