# Author Correction: Age related non-type 2 inflammation and its association with treatment outcome in patients with chronic rhinosinusitis with nasal polyp in Korea

**DOI:** 10.1038/s41598-023-30110-3

**Published:** 2023-02-17

**Authors:** Jeong-Whun Kim, Tae-Bin Won, Hyunjun Woo, Seung Koo Yang, Chayakoen Phannikul, Seung Ah Ko, Hyojin Kim, Chae‑Seo Rhee, Sung-Woo Cho

**Affiliations:** 1 Seoul National University College of Medicine, 82 Gumi-ro 173beon-gil, Bundang-gu, Seongnam, 13620 Korea; 2 Seoul National University College of Medicine, Seoul, Korea; 3 Seoul National University College of Medicine, Seongnam, South Korea

Correction to: *Scientific Reports* https://doi.org/10.1038/s41598-022-05614-z, published online 31 January 2022

The original version of this Article contained an error in the spelling of the author Jeong-Whun Kim which was incorrectly given as Jeong-Whum Kim.

The original Article has been corrected.

