# Supplementary material for: Age related non-type 2 inflammation and its association with treatment outcome in patients with chronic rhinosinusitis with nasal polyp in Korea
Source: Sci Rep. 2022 Jan 31;12:1671. doi: 10.1038/s41598-022-05614-z (PMC8803874; doi:10.1038/s41598-022-05614-z)
Supplement: Supplementary file 1 — Supplementary Information 1. [file 41598_2022_5614_MOESM1_ESM.pptx]

## Slide 1
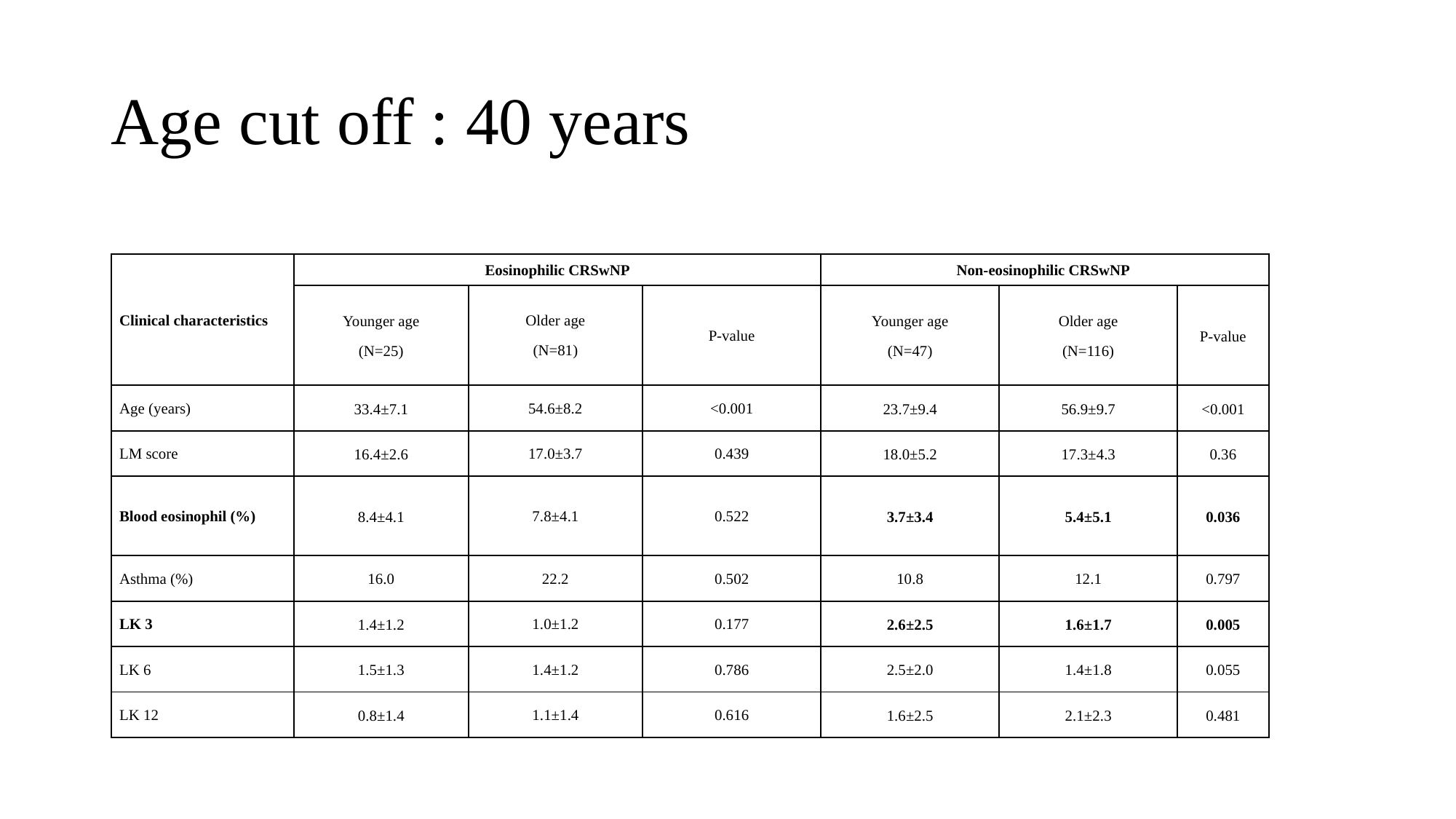

# Age cut off : 40 years
| Clinical characteristics | Eosinophilic CRSwNP | | | Non-eosinophilic CRSwNP | | |
| --- | --- | --- | --- | --- | --- | --- |
| | Younger age (N=25) | Older age (N=81) | P-value | Younger age (N=47) | Older age (N=116) | P-value |
| Age (years) | 33.4±7.1 | 54.6±8.2 | <0.001 | 23.7±9.4 | 56.9±9.7 | <0.001 |
| LM score | 16.4±2.6 | 17.0±3.7 | 0.439 | 18.0±5.2 | 17.3±4.3 | 0.36 |
| Blood eosinophil (%) | 8.4±4.1 | 7.8±4.1 | 0.522 | 3.7±3.4 | 5.4±5.1 | 0.036 |
| Asthma (%) | 16.0 | 22.2 | 0.502 | 10.8 | 12.1 | 0.797 |
| LK 3 | 1.4±1.2 | 1.0±1.2 | 0.177 | 2.6±2.5 | 1.6±1.7 | 0.005 |
| LK 6 | 1.5±1.3 | 1.4±1.2 | 0.786 | 2.5±2.0 | 1.4±1.8 | 0.055 |
| LK 12 | 0.8±1.4 | 1.1±1.4 | 0.616 | 1.6±2.5 | 2.1±2.3 | 0.481 |

## Slide 2
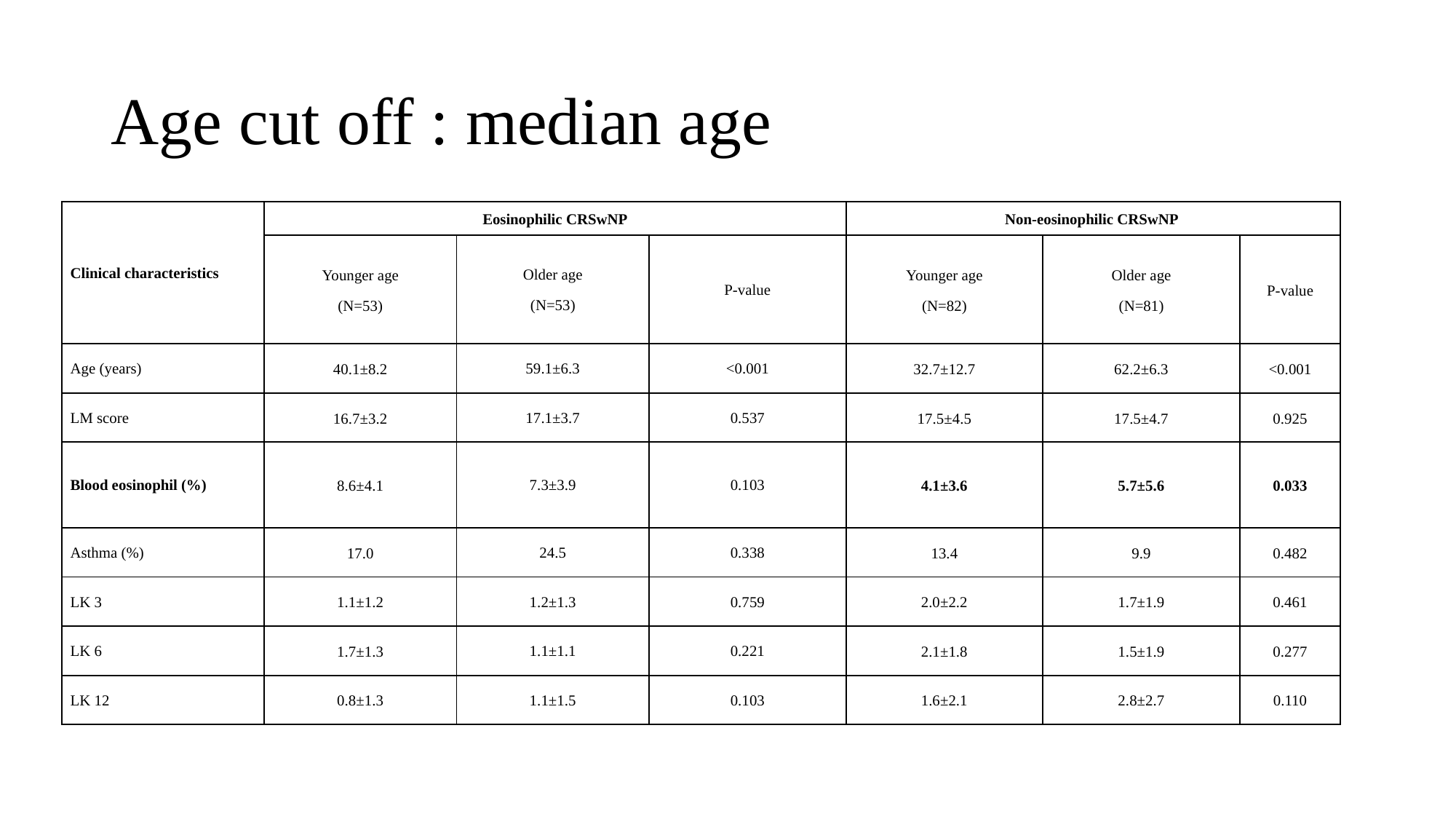

# Age cut off : median age
| Clinical characteristics | Eosinophilic CRSwNP | | | Non-eosinophilic CRSwNP | | |
| --- | --- | --- | --- | --- | --- | --- |
| | Younger age (N=53) | Older age (N=53) | P-value | Younger age (N=82) | Older age (N=81) | P-value |
| Age (years) | 40.1±8.2 | 59.1±6.3 | <0.001 | 32.7±12.7 | 62.2±6.3 | <0.001 |
| LM score | 16.7±3.2 | 17.1±3.7 | 0.537 | 17.5±4.5 | 17.5±4.7 | 0.925 |
| Blood eosinophil (%) | 8.6±4.1 | 7.3±3.9 | 0.103 | 4.1±3.6 | 5.7±5.6 | 0.033 |
| Asthma (%) | 17.0 | 24.5 | 0.338 | 13.4 | 9.9 | 0.482 |
| LK 3 | 1.1±1.2 | 1.2±1.3 | 0.759 | 2.0±2.2 | 1.7±1.9 | 0.461 |
| LK 6 | 1.7±1.3 | 1.1±1.1 | 0.221 | 2.1±1.8 | 1.5±1.9 | 0.277 |
| LK 12 | 0.8±1.3 | 1.1±1.5 | 0.103 | 1.6±2.1 | 2.8±2.7 | 0.110 |

## Slide 3
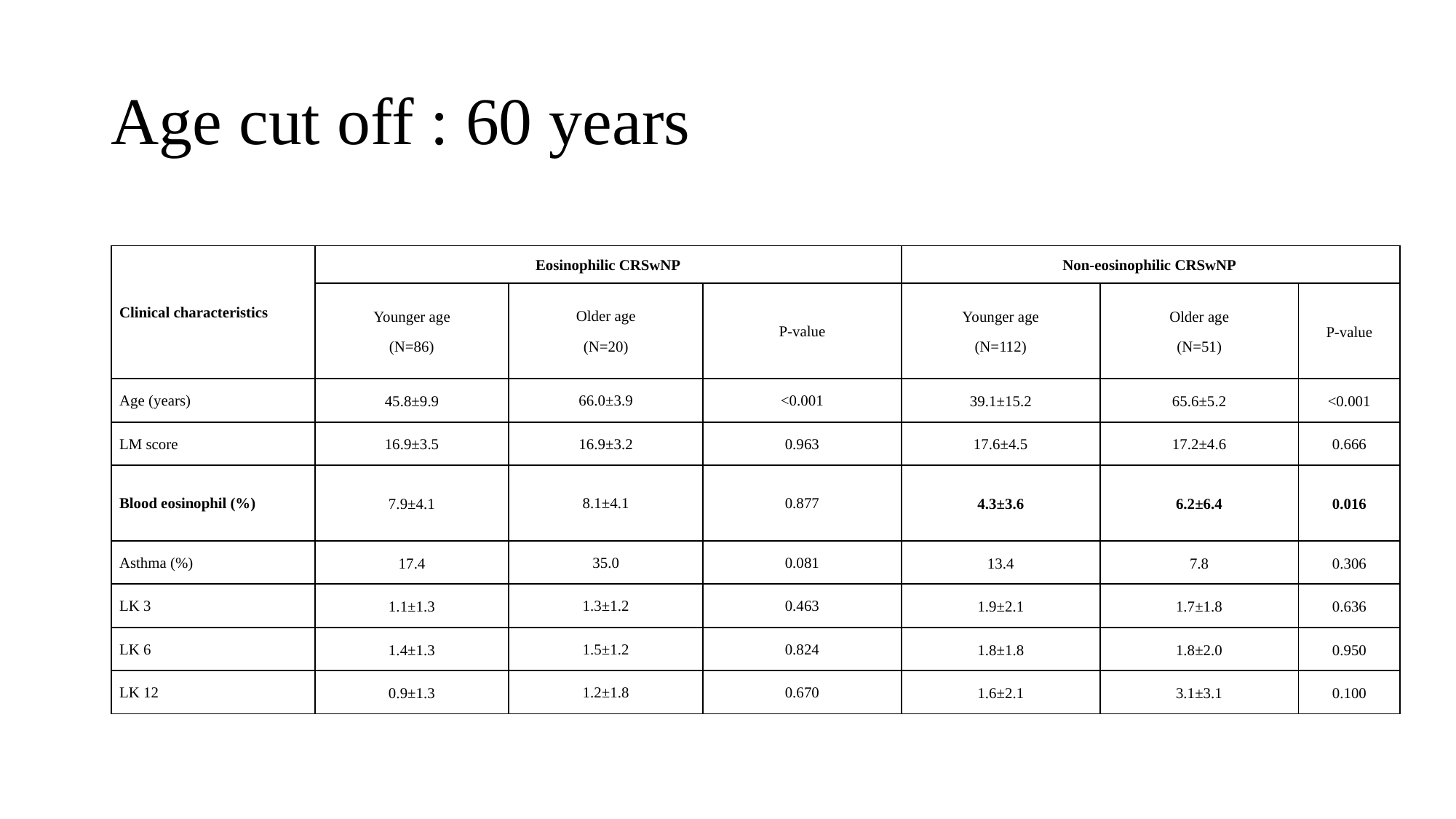

# Age cut off : 60 years
| Clinical characteristics | Eosinophilic CRSwNP | | | Non-eosinophilic CRSwNP | | |
| --- | --- | --- | --- | --- | --- | --- |
| | Younger age (N=86) | Older age (N=20) | P-value | Younger age (N=112) | Older age (N=51) | P-value |
| Age (years) | 45.8±9.9 | 66.0±3.9 | <0.001 | 39.1±15.2 | 65.6±5.2 | <0.001 |
| LM score | 16.9±3.5 | 16.9±3.2 | 0.963 | 17.6±4.5 | 17.2±4.6 | 0.666 |
| Blood eosinophil (%) | 7.9±4.1 | 8.1±4.1 | 0.877 | 4.3±3.6 | 6.2±6.4 | 0.016 |
| Asthma (%) | 17.4 | 35.0 | 0.081 | 13.4 | 7.8 | 0.306 |
| LK 3 | 1.1±1.3 | 1.3±1.2 | 0.463 | 1.9±2.1 | 1.7±1.8 | 0.636 |
| LK 6 | 1.4±1.3 | 1.5±1.2 | 0.824 | 1.8±1.8 | 1.8±2.0 | 0.950 |
| LK 12 | 0.9±1.3 | 1.2±1.8 | 0.670 | 1.6±2.1 | 3.1±3.1 | 0.100 |

## Slide 4
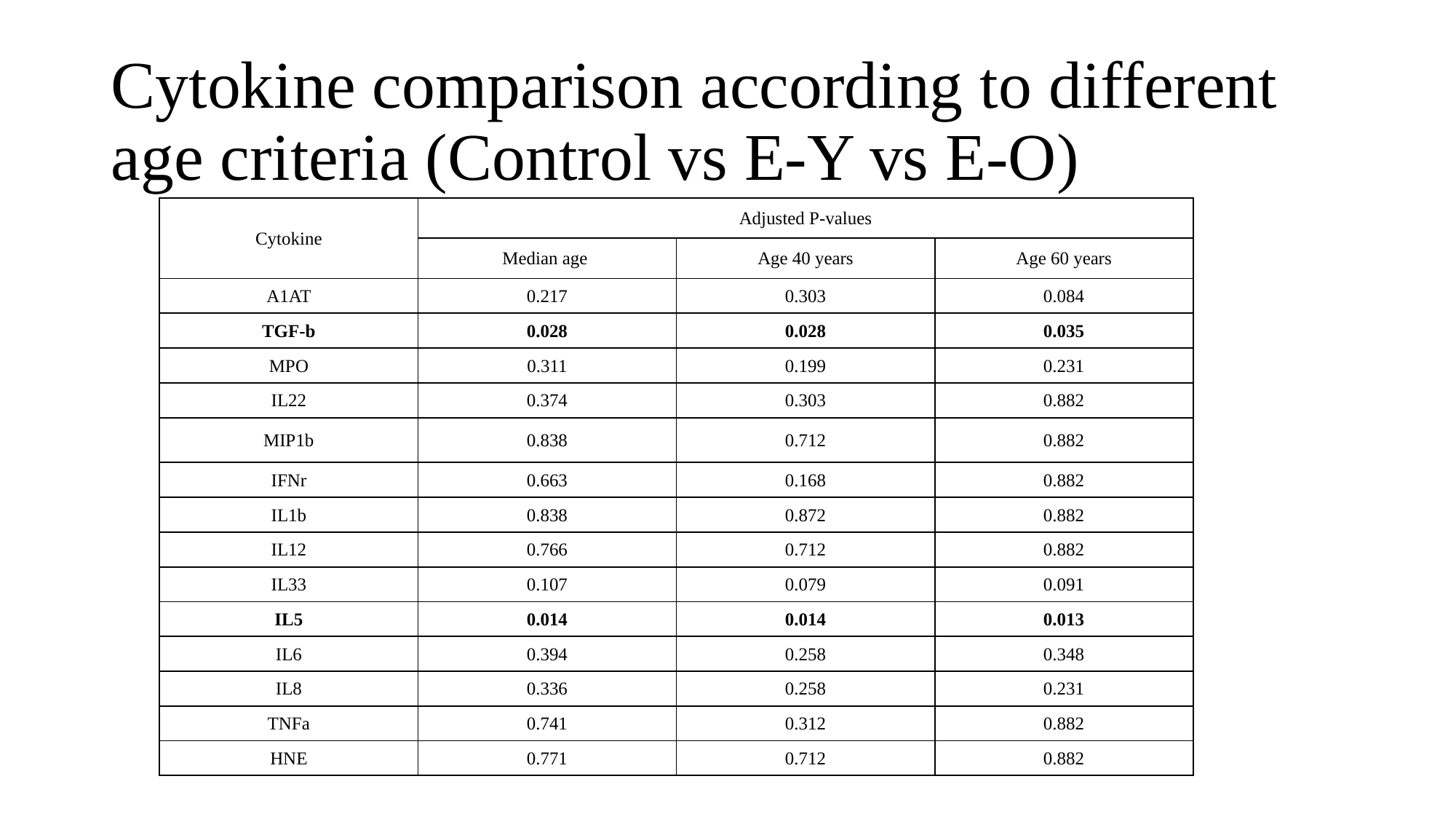

# Cytokine comparison according to different age criteria (Control vs E-Y vs E-O)
| Cytokine | Adjusted P-values | Adjusted p-values (40) | Adjusted p-values (60) |
| --- | --- | --- | --- |
| | Median age | Age 40 years | Age 60 years |
| A1AT | 0.217 | 0.303 | 0.084 |
| TGF-b | 0.028 | 0.028 | 0.035 |
| MPO | 0.311 | 0.199 | 0.231 |
| IL22 | 0.374 | 0.303 | 0.882 |
| MIP1b | 0.838 | 0.712 | 0.882 |
| IFNr | 0.663 | 0.168 | 0.882 |
| IL1b | 0.838 | 0.872 | 0.882 |
| IL12 | 0.766 | 0.712 | 0.882 |
| IL33 | 0.107 | 0.079 | 0.091 |
| IL5 | 0.014 | 0.014 | 0.013 |
| IL6 | 0.394 | 0.258 | 0.348 |
| IL8 | 0.336 | 0.258 | 0.231 |
| TNFa | 0.741 | 0.312 | 0.882 |
| HNE | 0.771 | 0.712 | 0.882 |

## Slide 5
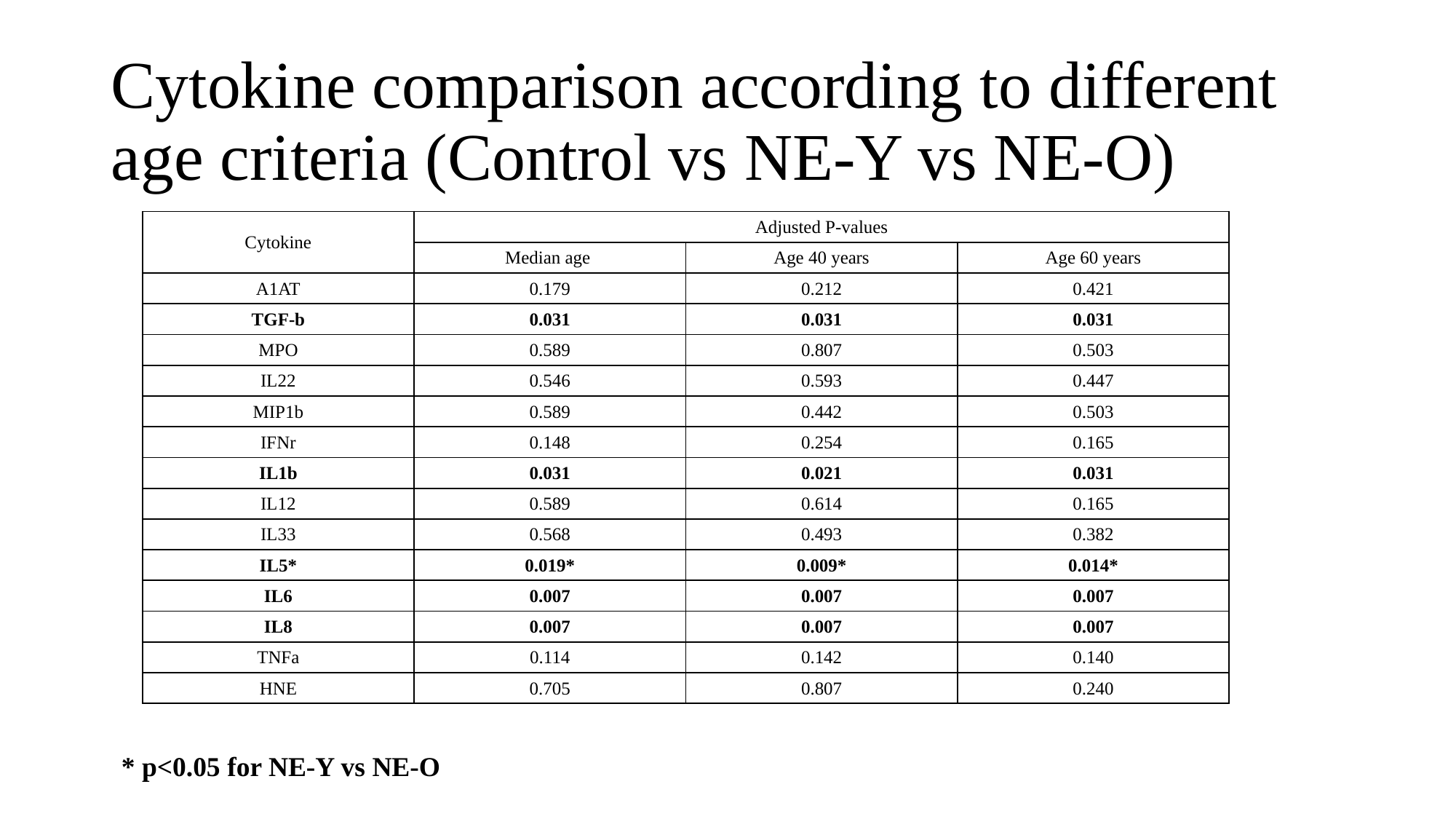

# Cytokine comparison according to different age criteria (Control vs NE-Y vs NE-O)
| Cytokine | Adjusted P-values | Age 40 years | Age 60 years |
| --- | --- | --- | --- |
| | Median age | Age 40 years | Age 60 years |
| A1AT | 0.179 | 0.212 | 0.421 |
| TGF-b | 0.031 | 0.031 | 0.031 |
| MPO | 0.589 | 0.807 | 0.503 |
| IL22 | 0.546 | 0.593 | 0.447 |
| MIP1b | 0.589 | 0.442 | 0.503 |
| IFNr | 0.148 | 0.254 | 0.165 |
| IL1b | 0.031 | 0.021 | 0.031 |
| IL12 | 0.589 | 0.614 | 0.165 |
| IL33 | 0.568 | 0.493 | 0.382 |
| IL5\* | 0.019\* | 0.009\* | 0.014\* |
| IL6 | 0.007 | 0.007 | 0.007 |
| IL8 | 0.007 | 0.007 | 0.007 |
| TNFa | 0.114 | 0.142 | 0.140 |
| HNE | 0.705 | 0.807 | 0.240 |
* p<0.05 for NE-Y vs NE-O
