# Supplementary material for: Age related non-type 2 inflammation and its association with treatment outcome in patients with chronic rhinosinusitis with nasal polyp in Korea
Source: Sci Rep. 2022 Jan 31;12:1671. doi: 10.1038/s41598-022-05614-z (PMC8803874; doi:10.1038/s41598-022-05614-z)
Supplement: Supplementary file 2 — Supplementary Information 2. [file 41598_2022_5614_MOESM2_ESM.docx]

| **Clinical characteristics** | **Control** | **Eosinophilic CRSwNP** | **Non-eosinophilic CRSwNP** | **P-value** |
| --- | --- | --- | --- | --- |
| N | 13 | 39 | 26 |  |
| Age (years) | 50.23±17.50 | 48.77±12.28 | 42.12±18.98 | 0.327 |
| M:F | 6:7 | 25:14 | 19:7 | 0.255 |
| LM score | 0.38±0.87 | 17.54±4.53 | 18.04±4.52 | <0.001 |
| Blood eosinophil (%) | 2.15±1.64 | 6.16±3.61* | 2.74±2.16 | <0.001 |
| Asthma | 0/13 | 11/39 | 3/26 | 0.042 |
| Smoking (%) | 23.1 | 45.9 | 54.2 | 0.187 |

**S1 Table. Patient characteristics of cohort 2**

Abbreviations. CRSwNP : Chronic rhinosinusitis with nasal polyp. LM : Lund Mackay, JESREC : The Japanese Epidemiology Survey of Refractory Eosinophilic Chronic Rhinosinusitus Study (JESREC). Results for continuous variables are presente d as mean with standard deviation. ***** P value < 0.05 for eosinophilic vs non-eosinophilic polyp by Kruskal-Wallis test with Dunn multiple comparison

**S2 Table .** Comparison of immunological characteristics of eosinophilic and non-eosinophilic CRSwNP. Values are presented as median with interquartile range (IQR). CRSwNP : Chronic rhinosinusitis with nasal polyp, A1AT: Alpha 1 antitrypsin, TGF: Transforming growth factor, MPO: myeloperoxidase, MIP: macrophage inflammatory protein, IFN: interferon, IL: interleukin, TNF: tumor necrosis factor, HNE: human neutrophil elastase. ***** P value < 0.05 for eosinophilic vs non-eosinophilic polyp by Kruskal-Wallis test with Dunn multiple comparison

| **Cytokine** | **Control** | **Eosinophilic CRSwNP** | **Non-eosinophilic**  **CRSwNP** | **P-value** |
| --- | --- | --- | --- | --- |
| A1AT (ng/ml) | 7.792(12.005) | 2.916(5.003) | 2.801 (9.858) | 0.287 |
| TGF-b (pg/ml) | 180.712(185.965) | 56.744(45.133) | 56.333 (46.525) | **0.007** |
| MPO (pg/ml) | 7550.833(38573.890) | 3072.324(11833.931) | 2851.735 (80305.920) | 0.089 |
| IL-22 (pg/ml) | 1.995 (7.038) | 0.567 (6.531) | 0.567 (3.570) | 0.734 |
| MIP1b (pg/ml) | 91.565 (68.393) | 90.321 (60.374) | 106.902 (57.331) | 0.176 |
| IFN-r (pg/ml) | 0.314 (3.059) | 1.351 (3.670) | 3.523 (7.360) | 0.206 |
| **IL-1b*(pg/ml)** | 0.001(0.431) | 0.001(0.338) | 2.600 (6.162) | **0.003** |
| IL-12 (pg/ml) | 0.118(0.000) | 0.118(0.000) | 0.118 (1.778) | 0.157 |
| IL-33 (pg/ml) | 1260.871 (768.614) | 743.841 (786.615) | 995.034 (865.853) | **0.022** |
| **IL-5*(pg/ml)** | 0.310(0.000) | 9.210(12.837) | 0.306 (0.393) | **0.003** |
| **IL-6*(pg/ml)** | 1.258(1.897) | 2.622(4.804) | 8.914 (25.981) | **0.004** |
| **IL-8*(pg/ml)** | 34.912 (18.872) | 50.048 (114.109) | 201.809 (397.316) | **0.003** |
| **TNF-α*(pg/ml)** | 0.655 (0.817) | 0.709 (0.800) | 1.213 (1.066) | **0.003** |
| HNE (pg/ml) | 8762.298 (15155.938) | 5468.316 (2004.485) | 5400.444 (4046.714) | 0.784 |

**S3 Table.** Comparison of immunological characteristics of eosinophilic polyps with age under 35 years (E-Y) and over 35 years (E-O) in cohort 1. Kruskal-Wallis test followed by post hoc analysis with Dunn multiple comparison showed no significant difference in cytokine levels between E-O and E-Y.

|  | **Control** | **E-Y** | **E-O** | **P-value*** |
| --- | --- | --- | --- | --- |
| A1AT (ng/ml) | 7.292  (12.005) | 5.388  (27.828) | 2.890  (4.385) | 0.325 |
| TGF-b (pg/ml) | 180.712  (185.965) | 34.941  (17.226-52.656) | 60.956  (44.722) | **0.021** |
| MPO (pg/ml) | 5770.833  (38573.890) | 9641.940  (35925.504) | 1556.867  (10056.683) | 0.102 |
| IL-22 (pg/ml) | 1.995  (7.038) | 0.567  (0.000) | 0.567  (6.872) | 0.543 |
| MIP1b (pg/ml) | 91.565  (68.393) | 104.554  (87.840-121.268) | 92.802  (68.746) | 0.824 |
| IFN-r (pg/ml) | 0.314  (3.059) | 0.314  (0.000) | 1.895  (3.904) | 0.347 |
| IL-1b (pg/ml) | 0.001  (0.431) | 0.001  (0.285) | 0.001  (0.431) | 0.861 |
| IL-12 (pg/ml) | 0.118  (0.000) | 0.118  (0.000) | 0.118  (0.000) | 0.791 |
| IL-33 (pg/ml) | 1260.871  (768.614) | 462.840  (213.391-712.289) | 775.392  (793.363) | 0.084 |
| IL-5 (pg/ml) | 0.306  (0.000) | 7.934  (8.455) | 10.113  (13.911) | **0.014** |
| IL-6 (pg/ml) | 1.258(  1.897) | 1.215  (11.461) | 2.786  (5.678) | 0.328 |
| IL-8 (pg/ml) | 34.912  (18.872) | 111.946  (10.551-213.340) | 52.704  (107.638) | 0.328 |
| TNF-a (pg/ml) | 0.655  (0.817) | 0.348  (0.238) | 0.731  (0.839) | 0.355 |
| HNE (ng/ml) | 8762.298  (15155.938) | 5300.564  (5132.812-5468.316) | 6039.359  (2292.158) | 0.791 |

Values are presented as median with interquartile range (IQR) or range. A1AT: Alpha 1 antitrypsin, TGF: Transforming growth factor, MPO: myeloperoxidase, MIP: macrophage inflammatory protein, IFN: interferon, IL: interleukin, TNF: tumor necrosis factor, HNE: human neutrophil elastase. * by Kruskal-Wallis test. None of the cytokines were significantly different between E-Y and E-O after Dunn’s post hoc analysis.

| **Polyp type** | **A1AT** | **TFG-ß** | **MPO** | **IL22** | **MIP1-ß** | **IFN-γ** | **IL1-ß** | **IL12** | **IL33** | **IL5** | **IL6** | **IL8** | **TNF-α** | **HNE** |
| --- | --- | --- | --- | --- | --- | --- | --- | --- | --- | --- | --- | --- | --- | --- |
| **Eosinophilic** | -0.226  (0.166) | 0.167  (0.361) | 0.015  (0.929) | 0.274  (0.129) | -0.160  (0.383) | 0.170  (0.353) | 0.051  (0.757) | -0.095  (0.606) | 0.088  (0.633) | -0.065  (0.695) | 0.202  (0.217) | 0.215  (0.238) | 0.243  (0.137) | 0.069  (0.722) |
| **Non-eosinophilic** | 0.314  (0.118) | 0.057  (0.780) | 0.072  (0.727) | 0.203  (0.321) | 0.140  (0.496) | 0.274  (0.175) | -0.101  (0.624) | -0.321  (0.110) | 0.139  (0.499) | **0.523^*^**  **(0.006)** | -0.046  (0.824) | -0.042  (0.837) | -0.077  (0.710) | 0.209  (0.315) |

**S4 Table. Correlation between tissue cytokine levels and age according to eosinophilic and non-eosinophilic polyp. Spearman’s correlation coefficients along with p-value (brackets) are described.** A1AT: Alpha 1 antitrypsin, TGF: Transforming growth factor, MPO: myeloperoxidase, MIP: macrophage inflammatory protein, IFN: interferon, IL: interleukin, TNF: tumor necrosis factor, HNE: human neutrophil elastase. ***** P value < 0.05
